# In Situ TEM Study of Structural Changes in Na-β″-Alumina Using Electron Beam Irradiation

**DOI:** 10.3390/ma15072663

**Published:** 2022-04-05

**Authors:** Sung-Dae Kim, Young-Woon Kim

**Affiliations:** 1 Advanced Metals Division, Korea Institute of Materials Science, 797 Changwondaero, Changwon 51508, Korea; 2 Department of Materials Science and Engineering, Seoul National University, 1 Gwanak-ro, Gwanak-gu, Seoul 151-744, Korea; sdkim57v@gmail.com

**Keywords:** Na-β″-alumina, ion conduction, electron beam irradiation, transmission electron microscopy

## Abstract

Real-time structural changes in Na-β″-alumina were observed in situ using transmission electron microscopy (TEM) with electron beam irradiation. Na-β″-alumina has been widely investigated as a solid electrolyte material for sodium–sulfur secondary batteries owing to its high ionic conductivity. This high conductivity is known to be due to the Na^+^ ions on the loosely packed conduction planes of Na-β″-alumina. In the present study, we acquired real-time videos of the generation of spinel blocks caused by the conduction of Na^+^ ions. In addition, by observing Na extraction during electron beam irradiation, we experimentally confirmed that spinel block generation originates from the Na^+^ ion conduction, which has been a subject of recent debate.

## 1. Introduction

Compared with liquid-electrolyte-based batteries, all-solid-state batteries with a ceramic electrolyte can guarantee higher safety and superior energy storage capability [1,2]. Lithium-ion batteries have become one of the most widely used energy storage technologies [3,4]. However, the limited resources and high cost of lithium have encouraged research on alternatives [5] such as batteries based on sodium, which has abundant resources and a low cost [6,7,8,9,10]. Na-β″-alumina has been widely investigated as a solid electrolyte for sodium–sulfur secondary batteries because of its high ionic conductivity, high density, and excellent stability against sodium metal [11,12,13]. The high mobility of Na^+^ ions in the conduction plane of Na-β″-alumina stems from the large number of possible Na^+^ sites between its spinel blocks [14,15,16]. With the chemical and structural analysis, the structure of Na β″-alumina was revealed, where Na ions are loosely linked between defective alumina spinels. The spinel-type blocks match the cubic structure of MgAl_2_O_4_ [12,17,18]. The conduction plane is composed of loosely packed O and Na ions, where the free Na ions are free to move. Chemical analysis and diffraction studies revealed that Na ions are present in the conduction plane at three candidate sites, such as Beevers-Ross (BR), anti-Beevers-Ross (aBR), and mid oxygen (mO). However, potential applications of Na-β″-alumina are limited by the certain degradation of its ionic conductivity with time, which is related to structural changes caused by the loss of Na^+^ ions from the positive electrode side [16,19]. Therefore, numerous studies have analyzed the atomic structure of Na-β″-alumina. In particular, such structural changes in Na-β″-alumina have been investigated using transmission electron microscopy (TEM) with electron beam irradiation because this accelerated beam can easily activate the Na ions on the conduction planes in β″-alumina [15,19,20,21,22]. For example, Bovin [19] carefully irradiated Na-β″-alumina with an electron beam and observed the growth of the spinel defect blocks via the conduction of Na^+^ ions caused by the electron beam. Since this study, electron-beam-induced structural changes (or Na ion conduction, or spinel block generation) in Na-β″-alumina have been intensively researched [15,19,20,21,22,23]. However, because most of the earlier investigations examined these structural changes in a post-mortem manner, they have not been quantitatively analyzed, and the ionic conduction dynamics have not been clearly visualized. In the present study, we used real-time in situ observations of the structural changes caused by electron beam irradiation in TEM to revisit Na-β″-alumina.

## 2. Materials and Methods

First, 6.5–9.2 wt% Na_2_CO_3_, 0.6–0.8 wt% Li_2_O, and 89.9 wt% Al_2_O_3_ with specific surface areas of 18–23 m^2^/g were used to produce the Li-stabilized Na-β″-alumina ceramic. The powders were isostatically pressed into disks at 350 MPa, followed by high-temperature sintering at 1400 °C with a heating rate of 150 °C in hermetically sealed platinum containers. The sintered Na-β″-alumina ceramics were then mechanically crushed into particles having cleaved edge regions that can be transparent to the electron beam. The phase identification of powders was performed by X-ray diffraction (XRD) using Cu Kα1 radiation (λ = 1.54056 Å). The crushed particles were dispersed on TEM mesh grids with a carbon support film. Structural changes in the lattice of the Na-β″-alumina were observed in real time using a field-emission transmission electron microscope (JEM-2100F, JEOL Ltd., Tokyo, Japan) at an acceleration voltage of 200 keV. Real-time recordings of these structural changes were acquired in situ using a charge-coupled device (CCD) camera at a recording rate of 30 frames per second. All of the in situ movies were acquired under electron beam dose of 6.5 A/m^2^.

## 3. Results

**Figure 1:**
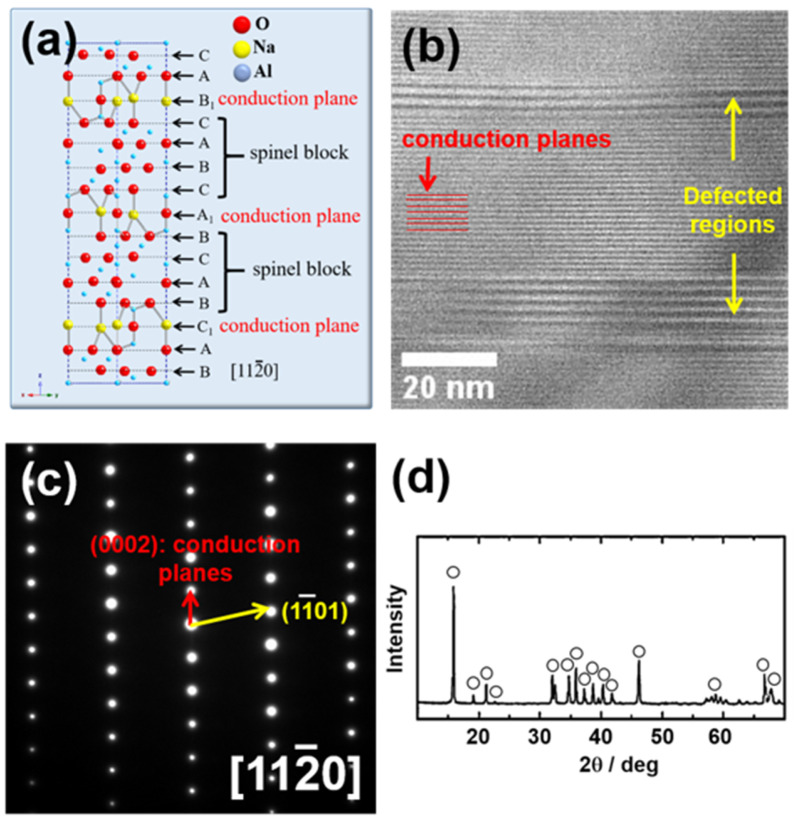
(**a**) Atomic structure of Na-β″-alumina viewed in the $\left[11\bar{2}0\right]$ direction, (**b**) bright-field (BF) TEM micrograph showing the lattice of conduction planes with faulted regions, selected area diffraction pattern (SADP) with a zone axis of $\left[11\bar{2}0\right]$, (**c**) selected area diffraction pattern (SADP) with a zone axis of $\left[11\bar{2}0\right]$, (**d**) XRD pattern of the Na-β″-alumina powders.

Figure 1a presents crystallographic information on Na-β″-alumina. Na-β″-alumina is composed of spinel blocks inserted between loosely packed conduction planes perpendicular to the c-axis [14,16,20]. This layered structure is visualized in Figure 1b, wherein the conduction planes appear as horizontal lattices. β″-alumina materials have a rhombohedral structure, as confirmed by the electron diffraction pattern in Figure 1c. Figure 1d shows the XRD result of the Na-β″-alumina powders. In Figure 1b, a faulted region appears in which some of the conduction layers are missing. Because Na ions are located in the conduction planes, it can be deduced that Na^+^ ions are ejected from the conduction planes. Conducting ions in β″-alumina are loosely linked to the adjacent spinel blocks, so they are mobile on these planes, which makes these materials an effective solid electrolyte for ionic conduction. Indeed, such ionic conduction could be activated by irradiation with the electron beam used in TEM; in other words, the beam electrically activates the loosely packed Na^+^ ions on the conduction plane, which then diffused out of this plane. Subsequently, this outward diffusion generates faulted layers where spinel blocks grow owing to the conduction plane removal.

**Figure 2:**
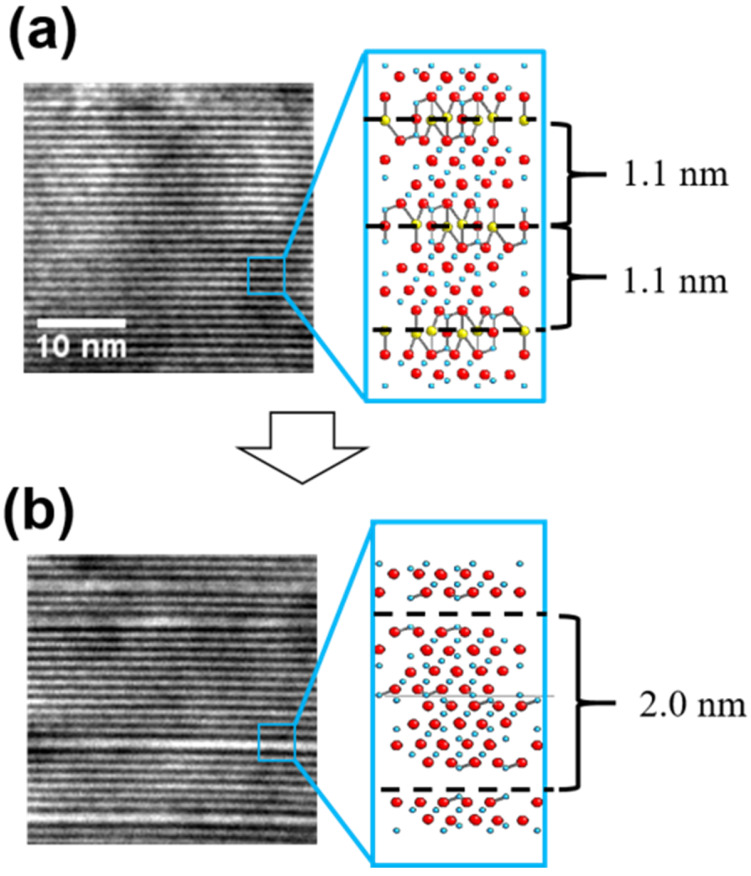
BF-TEM images and their corresponding atomic structures for (**a**) pristine and (**b**) faulted Na-β″-alumina in the of $\left[11\bar{2}0\right]$ viewing direction.

Figure 2 more clearly demonstrates the structural changes caused by the electron-beam-induced Na^+^ ion conduction. Figure 2a shows Na-β″-alumina in the pristine state from a viewing direction parallel to the basal plane of the β″-alumina, which makes the Na-containing conduction planes appear black lines. The interspacing of the conduction planes was measured to be 1.1 nm. After electron beam irradiation (Figure 2b), Na^+^ ions appear to have diffused out from some of the conduction layers, leaving grown spinel blocks. Consequently, the spacing between the conduction layers increased to 2.0 nm in the faulted region.

**Figure 3:**
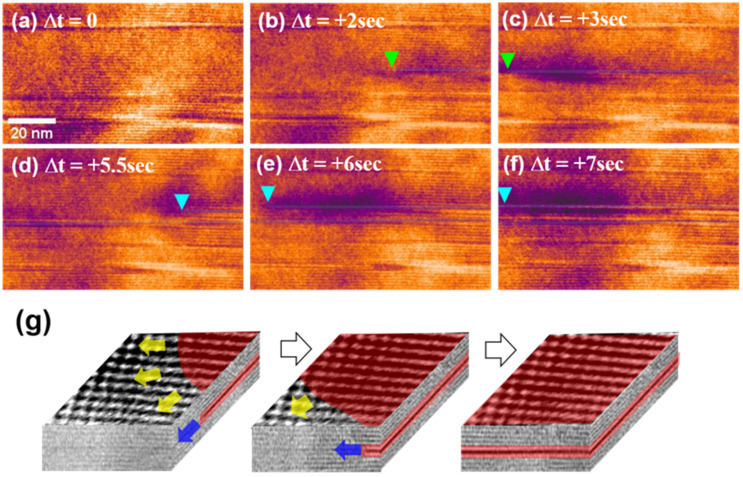
(**a**–**f**) Sequential BF-TEM images showing the growth of faulted regions caused by Na ion conduction. (**g**) Three-dimensional representation (perspective diagrams) of the spinel block generation. The top view images were extracted from the Movie S2 (viewing direction: [0001]). The front and side view images were taken from the Movie S3 (viewing direction: $\langle 11\bar{2}0\rangle$) of the spinel blocks generation.

Figure 3a–f shows sequential bright-field (BF)-TEM images of the growth of the faulted region. These images were extracted from real-time observations of the electron-beam-induced Na ion conduction, provided in the Supplementary Materials as Movie S1. Figure 3g schematically depicts the generation of the faulted layer via Na ion conduction. Na conduction is visualized in the direction normal to the conduction layers (i.e., [0001]) in real time in Movie S2. When viewed in this direction, waves seem to sweep across the sample surface.

**Figure 4:**
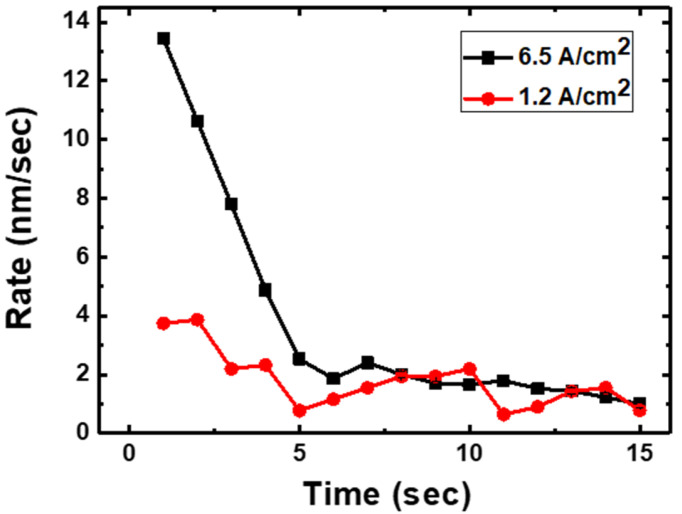
Growth rates of the spinel blocks with different electron beam doses.

Figure 4 shows the growth rates of the spinel blocks under different electron beam doses. Under a high dose (6.5 A/cm^2^), the initial speed of the spinel growth front was measured to be 13.6 ± 0.8 nm/s, but it decreased to a saturated value below 2 nm/s. When the electron beam dose was reduced to 1.2 A/cm^2^, the initial spinel growth was relatively sluggish at 4.1 nm ± 0.3 nm/s, but this speed also saturated at approximately 2 nm/s. The origin of the electron-beam-induced spinel block generation in Na-β″-alumina is currently under debate. Specifically, some reports claimed that the Na^+^ ions on the conduction planes are thermally activated by the accelerated electron beam in TEM [19], whereas others maintained that the breakdown of the charge neutrality caused by the electron beam leads to Na^+^ ion conduction [14,15]. The time-dependent decrease in the growth rates of the spinel blocks (Figure 4) supports the latter claim, i.e., continuous irradiation with the electron beam recovers the charge neutrality of the observed region, thereby reducing the activation energy for Na^+^ ion migration.

**Figure 5:**
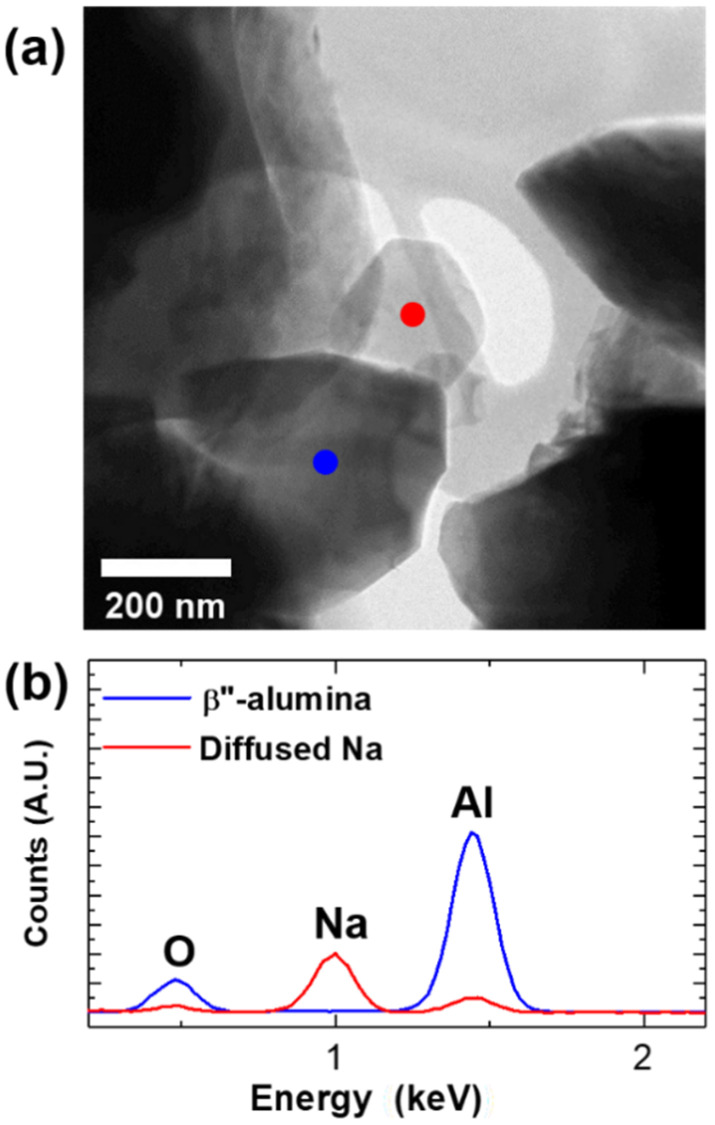
(**a**) Low-magnification BF-TEM image showing Na extraction from the Na-β″-alumina by electron beam illumination, (**b**) energy-dispersive spectroscopy (EDS) results obtained from the β″-alumina (blue dot) and extracted Na (red dot).

The diffusion of the Na^+^ ions was confirmed by X-ray energy-dispersive spectroscopy (EDS), as shown in Figure 5. As the Na-β″-alumina sample was irradiated by the accelerated electron beam in TEM, a liquid-like droplet (marked by a red dot) grew on the sample surface, as shown in Figure 5a. The EDS elemental analysis confirmed that the drop was extracted Na ions (red line), as shown in Figure 5b. Because crystalline metals tend to equilibrate toward the morphology with the lowest total surface energy [24], the extracted Na drop eventually formed into a polygonal shape. The Na ion extraction was recorded in Movie S4. Even the intensity is relatively lower than that of the Na peak, there are Al and O peaks in the point EDS result. The detection of Al and O in the polygon might be due to the interaction volume of the electron beam. However, it is hard to understand the exact origin of the detection of the Al and O element in the extracted Na-rich polygon. Therefore, a deep investigation of the extraction of Na droplets by electron beam irradiation is needed in a future study.

**Figure 6:**
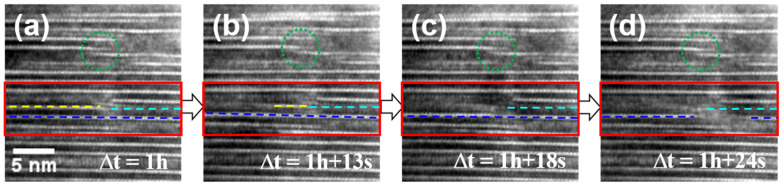
Sequential TEM images showing the inter-growth of faulted regions across conduction layers.

Notably, this study presents the first experimental evidence of Na extraction from Na-β″-alumina. After 1 h of electron beam irradiation, the faulted area in Na-β″-alumina increased to ~30%, as shown in Figure 6a. A previous article [25] reported that the growth of spinel blocks owing to Na^+^ ion conduction is frequently impinged by other pre-existing spinel blocks, thereby degrading the ionic conductivity of Na-β″-alumina. In the present study, the growth of spinel blocks was also impinged by long-term electron beam irradiation (as indicated by green dotted circles in Figure 6). In addition, as highlighted by red rectangles in Figure 6a–d and shown in Movie S5, a new spinel block can be activated at the adjacent layer (blue dashed lines in Figure 6) when a spinel block that is growing by consuming the conduction layer (yellow dashed lines in Figure 6a,b) encounters a pre-existing spinel block (cyan dotted lines in Figure 6).

## 4. Conclusions

Structural changes in Na-β″-alumina caused by electron beam irradiation in TEM were investigated in situ, and the generation of spinel blocks caused by the conduction of the Na^+^ ions was observed in real time. In addition, electron-beam-induced Na^+^ ion conduction irradiation was evidenced by an EDS elemental analysis, which confirmed that the metal was extracted. A quantitative analysis of the spinel block growth rate supported that the breakdown of charge neutrality by the electron beam led to Na^+^ ion conduction. Finally, although Na^+^ ion conduction was exhausted after long-term electron beam irradiation, the formation of a new spinel block could be activated when a growing spinel block encountered a pre-existing spinel block.

## Data Availability

Not applicable.

## Supplementary Materials

The following supporting information can be downloaded at: https://www.mdpi.com/article/10.3390/ma15072663/s1, Movies S1–S5.
